# First Report of Multidrug-Resistant *Staphylococcus sciuri* Isolated from the Urinary Bladder of a Domestic Rabbit in Romania: A Case Study

**DOI:** 10.3390/antibiotics14111089

**Published:** 2025-10-29

**Authors:** Bogdan Florea, Doru Morar, Cristina Văduva, Florin Simiz, Simina Velescu, Corina Kracunovic, Vlad Iorgoni, Paula Nistor, Janos Degi, Ionica Iancu, Viorel Herman, Alexandra Pocinoc, Eugenia Dumitrescu

**Affiliations:** 1 Department of Internal Medicine, University of Life Sciences “King Mihai I” from Timisoara, 300645 Timisoara, Romania; bogdan-alexandru.florea.fmv@usvt.ro (B.F.); florinsimiz@usvt.ro (F.S.); simina.velescu@usvt.ro (S.V.); corina.kracunovic@usvt.ro (C.K.); 2 Department of Infectious Diseases and Preventive Medicine, University of Life Sciences “King Mihai I” from Timisoara, 300645 Timisoara, Romania; vlad.iorgoni@usvt.ro (V.I.); paula.nistor@usvt.ro (P.N.); janosdegi@usvt.ro (J.D.); ionica.iancu@usvt.ro (I.I.); viorelherman@usvt.ro (V.H.); 3 Department of Parasitology, University of Life Sciences “King Mihai I” from Timisoara, 300645 Timisoara, Romania; alexandra.pocinoc@usvt.ro; 4 Department of Pharmacy and Pharmacology, University of Life Sciences “King Mihai I” from Timisoara, 300645 Timisoara, Romania; eugeniadumitrescu@usvt.ro

**Keywords:** *Staphylococcus sciuri*, multidrug resistance, domestic rabbit, urinary tract infection, antimicrobial susceptibility, MALDI-TOF MS, zoonotic potential

## Abstract

**Background/Objectives**: *Staphylococcus sciuri*, traditionally regarded as a commensal organism in animals and the environment, is increasingly recognized as a potential opportunistic pathogen with zoonotic significance. Its genomic reservoir of methicillin resistance homologues further raises concern regarding its role in antimicrobial resistance dissemination. This study describes the first documented case of *S. sciuri* isolated from the urinary bladder of a domestic rabbit (*Oryctolagus cuniculus*) in Romania, emphasizing its clinical relevance and antimicrobial profile. **Methods**: A seven-year-old intact female rabbit presenting with apathy, dysuria, and hematuria underwent clinical evaluation, ultrasonography, and cystocentesis. The aspirated intravesical content was subjected to bacterial culture, MALDI-TOF MS identification, and antimicrobial susceptibility testing via the VITEK 2 system. **Results**: Pure colonies of Gram-positive cocci were identified as *S. sciuri* with high confidence. Antimicrobial susceptibility testing revealed susceptibility to β-lactams, aminoglycosides, glycopeptides, linezolid, rifampicin, fusidic acid, tigecycline, and trimethoprim-sulfamethoxazole, while resistance was observed against fluoroquinolones, macrolides, lincosamides, and tetracycline, indicating a multidrug-resistant phenotype. Treatment with trimethoprim-sulfamethoxazole combined with ultrasound-guided bladder lavage and supportive therapy resulted in complete clinical recovery within 10 days. **Conclusions**: This case highlights the pathogenic potential of *S. sciuri* in domestic rabbits and its capacity to exhibit multidrug resistance. The findings underscore the necessity of including rabbits in antimicrobial resistance surveillance programs and reinforce the importance of culture and sensitivity testing in guiding the therapeutic management of exotic companion animals.

## 1. Introduction

The genus *Staphylococcus* was first described in 1880, and nearly a century later, *Staphylococcus sciuri* was identified by Kloos and colleagues in 1976 as a common bacterial species exhibiting remarkable ecological adaptability [1]. This species was subsequently classified within the *S. sciuri* group, alongside closely related taxa such as *S. lentus, S. vitulinus, S. fleurettii,* and *S. stepanovicii* [2,3]. Members of this group are typically coagulase-negative and novobiocin-resistant, regarded as commensals primarily associated with animals, although they can also be isolated from environmental sources such as dust and soil [4].

Unlike *Staphylococcus aureus*, which is well known for its clinical significance, *S. sciuri* group members are most frequently isolated from healthy animals [5,6,7]. However, this group is of increasing interest due to the presence of chromosomal homologues of the methicillin resistance gene mecA [8,9]. Although these variants do not directly confer a methicillin-resistant phenotype, they may act as a genetic reservoir for the mecA gene located on the staphylococcal cassette chromosome (SCCmec), a mobile genetic element implicated in methicillin resistance in *S. aureus*. To date, at least fourteen distinct SCCmec types have been identified [10,11].

Furthermore, *S. sciuri* has been implicated in clinical infections in both animals and humans, highlighting its potential zoonotic relevance [12]. Staphylococci are widely distributed across various ecological niches and are frequently found colonizing the skin and mucous membranes of humans, domestic animals, and birds. They can cause a wide spectrum of infections, ranging from mild skin conditions to severe, potentially life-threatening diseases such as endocarditis, pneumonia, and septicemia. Their clinical impact is significantly enhanced by their ability to develop resistance to multiple classes of antimicrobial agents [13,14].

In this context, it is crucial to investigate antimicrobial resistance in *Staphylococcus* spp. isolated from animals, particularly domestic species that live in close contact with humans and may serve as reservoirs for antimicrobial resistance genes [15,16,17].

The domestic rabbit (*O. cuniculus*) is a widely distributed mammalian species, commonly raised for meat, for fur, and as a companion animal. In Romania, rabbit farming is practiced on a small to medium scale, and rabbits are also frequently kept as pets. Despite their growing importance in both agriculture and household settings, domestic rabbits remain under-represented in surveillance programs focused on antimicrobial resistance. However, as with other domestic animals, they can harbor bacterial strains with potential zoonotic and clinical implications.

Given the potential of domestic rabbits to carry resistant staphylococci, the present study aimed to (i) isolate and identify *Staphylococcus* species from the urinary bladder of a domestic rabbit (*O. cuniculus*) in Romania and (ii) determine the antimicrobial susceptibility profiles of the isolates.

## 2. Case Study

A seven-year-old, intact female domestic rabbit (*O. cuniculus*) was presented for veterinary consultation with a history of apathy lasting eight days. According to the owner, the rabbit exhibited markedly reduced feed intake during this period, with selective consumption of only small amounts of preferred food items over the last two days. In addition, hematuria and signs of dysuria were observed, raising suspicion of a lower urinary tract disorder.

On clinical examination, the rabbit weighed 2.7 kg, consistent with a slight reduction in body condition compared with its previously recorded weight. The oral cavity was examined thoroughly, and the dentition displayed no abnormalities, eliminating dental disease as a primary cause of reduced feed intake. General inspection revealed no other significant external abnormalities. However, abdominal palpation elicited marked discomfort, particularly in the caudal region, suggesting involvement of the urinary bladder. Mucous membranes were pink and capillary refill time was within normal limits, indicating preserved peripheral perfusion. Rectal temperature, heart rate, and respiratory rate were within reference intervals for the species.

**Figure 1:**
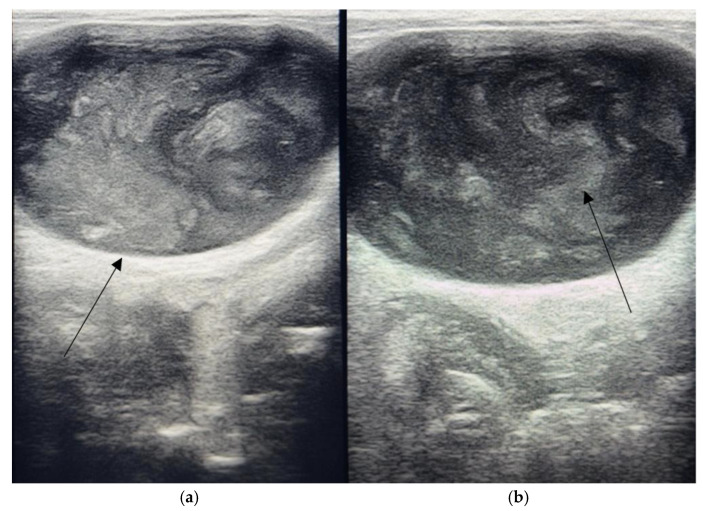
The ultrasonographic appearance of the urinary bladder in a rabbit naturally infected with *Staphylococcus sciuri*. The bladder wall exhibits increased echogenicity, indicative of chronic inflammatory infiltration and mucosal edema. (**a**) The intraluminal content is heterogeneously hyperechoic, containing suspended particulate material and flocculent debris consistent with inflammatory exudate, epithelial desquamation, and bacterial aggregates. (**b**) These sonographic features are characteristic of *bacterial cystitis* with advanced inflammatory remodeling of the bladder wall and accumulation of dense sediment within the lumen.

Given the clinical suspicion of urinary tract disease, abdominal ultrasonography was performed. The examination revealed normal echogenicity and morphology of the liver, spleen, kidneys, and gastrointestinal tract. In contrast, the urinary bladder demonstrated abnormal characteristics (Figure 1). Instead of a typical anechoic lumen, the bladder exhibited increased echogenicity and a heterogeneous intraluminal structure with the appearance of an irregularly defined mass-like structure. The wall thickness appeared within the normal range, but the echogenic material within the lumen raised concern for either severe cystitis with sedimentation, intravesical mass, or atypical exudative accumulation.

To further clarify the diagnosis, an ultrasound-guided cystocentesis was performed under gentle restraint. Unexpectedly, aspiration yielded a gelatinous, highly viscous material rather than the normal liquid urine. The aspirated substance was translucent with visible streaks of blood of jelly-like consistency. Such gross characteristics suggested a pathological process involving extensive proteinaceous exudation and cellular debris. The collected sample was divided into aliquots for cytological evaluation, bacterial culture, and antimicrobial susceptibility testing.

**Figure 2:**
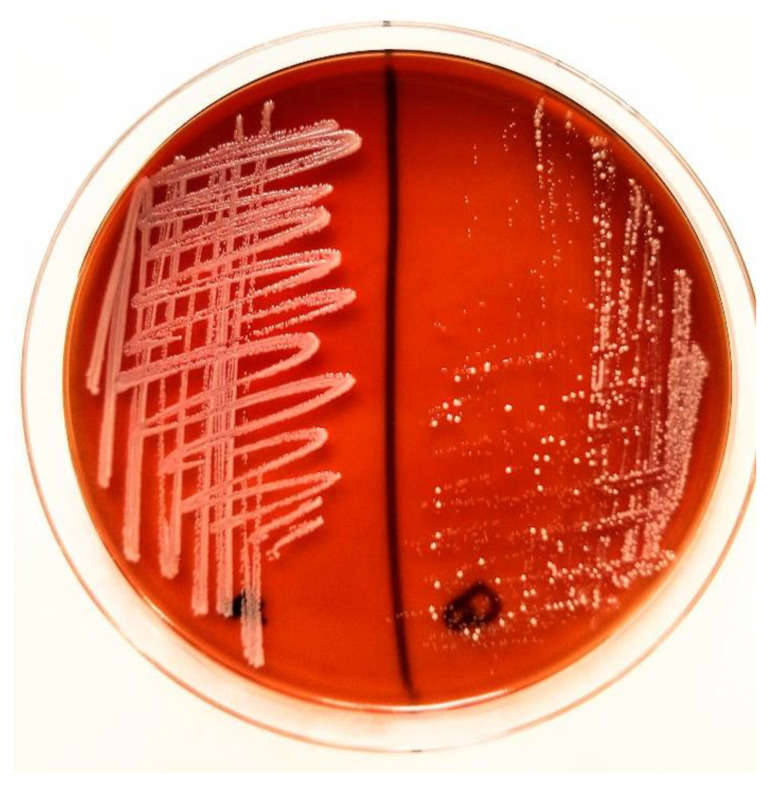
Growth of *Staphylococcus sciuri* on blood agar after incubation. Pure colonies developed, showing a whitish-gray, smooth morphology characteristic of staphylococcal species.

Bacterial culture was initiated by inoculating the specimen onto Columbia agar supplemented with 5% sheep blood and MacConkey agar to allow differential growth. The plates were incubated aerobically at 37 °C and inspected after 24 and 48 h. After incubation on Columbia agar supplemented with 5% sheep blood at 37 °C for 24 h, pure whitish-gray, smooth colonies developed, consistent with staphylococcal morphology (Figure 2). No growth was observed on MacConkey agar, consistent with the Gram-positive profile of the isolate. Gram staining confirmed Gram-positive cocci arranged predominantly in clusters.

For precise identification, matrix-assisted laser desorption/ionization time-of-flight mass spectrometry (MALDI-TOF MS) was employed. The isolate was conclusively identified as *Staphylococcus sciuri*. A comparison against reference strains in the Bruker MALDI Biotyper database confirmed. To confirm species identity, the 16S rRNA gene was amplified using universal primers 27F/1492R and sequenced (Macrogen Europe). The obtained sequence showed 99.7% identity with *Staphylococcus sciuri* ATCC 29062 (GenBank accession CP011134).

Antimicrobial susceptibility testing was carried out using the VITEK 2 automated system (bioMérieux, France). The minimum inhibitory concentration (MIC) values were determined for a broad spectrum of antimicrobials, grouped by pharmacological class. The isolate demonstrated susceptibility to all tested β-lactams, including penicillin (MIC ≤ 0.12 µg/mL) and oxacillin (MIC ≤ 0.25 µg/mL), as well as to aminoglycosides such as gentamicin (MIC ≤ 1 µg/mL). It also showed susceptibility to glycopeptides (vancomycin, MIC ≤ 1 µg/mL; teicoplanin, MIC ≤ 1 µg/mL), the oxazolidinone linezolid (MIC ≤ 1 µg/mL), and other agents including tigecycline (MIC ≤ 0.25 µg/mL), fusidic acid (MIC ≤ 1 µg/mL), rifampicin (MIC ≤ 0.5 µg/mL), and trimethoprim-sulfamethoxazole (MIC ≤ 0.5/9.5 µg/mL). Conversely, resistance was documented against fluoroquinolones—ciprofloxacin (MIC ≥ 4 µg/mL) and moxifloxacin (MIC ≥ 8 µg/mL)—as well as against macrolides and lincosamides, including erythromycin (MIC ≥ 8 µg/mL) and clindamycin (MIC ≥ 8 µg/mL). Resistance was also noted to tetracycline (MIC ≥ 16 µg/mL) [18].

Antimicrobial susceptibility testing was performed using the VITEK 2 Compact system (bioMérieux, France) with GP67 AST cards. Minimum inhibitory concentrations (MICs) were interpreted according to the Clinical and Laboratory Standards Institute (CLSI) M100, 34th edition (2024) guidelines. *Staphylococcus aureus* ATCC 29,213 and *Enterococcus faecalis* ATCC 29,212 were used as quality-control strains to validate assay performance. Antimicrobial susceptibility was evaluated across eight classes (β-lactams, aminoglycosides, glycopeptides, oxazolidinones, tetracyclines, macrolides, lincosamides, and fluoroquinolones). Resistance was present in four classes—fluoroquinolones (ciprofloxacin, moxifloxacin), macrolides (erythromycin), lincosamides (clindamycin), and tetracyclines (tetracyclines)—while the isolate remained susceptible to all tested β-lactams, aminoglycosides, glycopeptides, and linezolid. Multidrug resistance was defined as resistance to ≥3 antimicrobial classes [18].

These findings indicated a multidrug-resistance pattern affecting several major antibiotic classes, but with retained sensitivity to multiple therapeutic options of clinical relevance.

Based on the antibiogram results, antimicrobial therapy was initiated with trimethoprim-sulfamethoxazole, administered orally at 30 mg/kg twice daily for 10 consecutive days. In addition to systemic treatment, a therapeutic bladder lavage was performed under ultrasound guidance to remove the pathological intravesical content, which exhibited a gelatinous consistency incompatible with normal urine. Sterile isotonic saline solution was used for lavage until the aspirated fluid became macroscopically clear, facilitating both the mechanical removal of the exudative material and a reduction in bacterial load.

Supportive care included subcutaneous fluid therapy with Ringer’s lactate (20 mL/kg daily) to ensure adequate hydration, meloxicam (0.3 mg/kg orally, once daily) for analgesia and anti-inflammatory effect, and assisted enteral feeding with a high-fiber recovery formula to prevent gastrointestinal hypomotility. Probiotic supplementation was provided throughout treatment to counteract potential dysbiosis associated with antimicrobial administration.

The rabbit demonstrated marked clinical improvement within 72 h, with the normalization of feed intake and increased activity. Hematuria and dysuria were resolved by the fifth day of therapy. Follow-up ultrasonography on day seven confirmed a near-normal bladder lumen, with disappearance of the abnormal echogenic content previously observed. At the completion of the 10-day antimicrobial course, combined with lavage and supportive management, the rabbit exhibited complete recovery, with stable body weight, normal urination, and restored appetite.

This case highlights the pathogenic potential of *S. sciuri*, a species traditionally regarded as a commensal organism but increasingly recognized in veterinary and medical contexts as an opportunistic pathogen. The isolation of this organism from a normally sterile site, coupled with consistent clinical signs and successful treatment outcome, supports its role as the etiological agent in this infection. Furthermore, the demonstrated resistance profile underscores the necessity of culture and sensitivity testing in guiding therapeutic decisions in exotic companion animals, where empirical treatment may otherwise fail.

## 3. Discussion

In the present study, we report the isolation of *Staphylococcus sciuri* from the urinary bladder of a domestic rabbit (*O. cuniculus*) in Romania. Although the urinary bladder has traditionally been regarded as sterile, multiple human studies, using enhanced urine culture and metagenomics, have demonstrated a urinary microbiome with potential protective roles. In domestic rabbits, evidence remains scarce/emerging; therefore, recovery of a high-abundance organism from intravesical material in a compatible clinical context remains pathologically meaningful. [19,20,21]. Several members of the *S. sciuri* group harbor chromosomal *mecA* homologues and additional resistance determinants such as *tet*, *aac/aph*, *sal(A)*, *fexA*, and *blaZ*, which mediate resistance to β-lactams, tetracyclines, aminoglycosides, and lincosamides. Efflux-mediated macrolide resistance has also been reported in this species. These findings highlight its role as a genetic reservoir contributing to the horizontal transfer of antimicrobial resistance genes across staphylococci [22,23].

In this context, the presence of *S. sciuri* in domestic rabbits may indicate a previously under-recognized reservoir for antimicrobial resistance genes, especially in backyard or household animals that are in close and frequent contact with humans. This is particularly relevant in Romania and other Eastern European countries, where antimicrobial resistance surveillance data in rabbits and other minor companion or livestock species remain scarce. The potential for interspecies gene transfer and zoonotic transmission further underscores the importance of incorporating rabbits into antimicrobial resistance monitoring programs [24].

Additionally, although *S. sciuri* is generally regarded as a low-virulence species, it has been associated with a variety of clinical conditions, including urinary tract infections, wound infections, and bacteremia, particularly in immunocompromised hosts. The presence of this organism in the urinary tract of a rabbit may suggest either a subclinical infection or colonization of a normally sterile site, possibly facilitated by host or environmental factors [25,26].

Given the ability of *S. sciuri* to survive in diverse environments and its documented resistance to multiple antimicrobial agents, its isolation from a domestic animal warrants further investigation, particularly concerning its antimicrobial susceptibility profile and virulence potential. Although this study was limited to a single isolate, the findings support the growing body of evidence suggesting that non-aureus staphylococci (NAS) deserve more attention in both veterinary and public health contexts. Comparable clinical manifestations associated with multidrug-resistant E. coli have been documented in domestic rabbits and others animals from the same geographical region, suggesting that companion lagomorphs may serve as incidental hosts for diverse resistant bacterial pathogens [27,28,29,30].

Future studies should aim to expand sample sizes and geographic coverage and include the molecular characterization of resistance genes and virulence factors. Comparative genomic analysis with other *S. sciuri* isolates from animals and humans may also help clarify the evolutionary dynamics and epidemiological significance of this species.

## 4. Conclusions

*Staphylococcus sciuri*, although often considered a commensal organism, can act as an opportunistic pathogen in domestic rabbits, causing clinically significant urinary tract infections. The multidrug resistance profile observed in this isolate emphasizes the risk posed by non-aureus staphylococci as reservoirs of antimicrobial resistance genes with potential zoonotic implications. The successful outcome following targeted antimicrobial therapy and bladder lavage demonstrates the importance of diagnostic precision and individualized treatment in exotic companion animal medicine. Broader surveillance efforts are warranted to better assess the epidemiological and clinical significance of *S. sciuri* in rabbits and other domestic species.

These findings highlight the relevance of monitoring antimicrobial resistance in minor companion species within the One Health framework.

## Data Availability

The original contributions presented in this study are included in the article. Further inquiries can be directed to the corresponding author.
